# Supplementary material for: Cell sorting based on pulse shapes from angle resolved detection of scattered light
Source: Commun Biol. 2024 Aug 30;7:1063. doi: 10.1038/s42003-024-06759-5 (PMC11364749; doi:10.1038/s42003-024-06759-5)
Supplement: Supplementary file 2 — Supporting Information [file 42003_2024_6759_MOESM2_ESM.pdf]

Supporting Information:
Cell sorting based on pulse shapes from angle resolved detection of scattered light

Daniel Kage[1], Andrej Eirich[2], Kerstin Heinrich[1], Jenny Kirsch[1], Jan Popien[2], Alexander Wolf[1], Konrad v. Volkmann[2], Hyun-Dong Chang[1,3], Toralf Kaiser[1]

[1] German Rheumatology Research Center (DRFZ) - Flow Cytometry Core Facility, Charitéplatz 1 (Virchowweg 12), 10117 Berlin, Germany
[2] APE Angewandte Physik und Elektronik GmbH, Plauener Straße 163-165 / Haus N, 13053 Berlin, Germany
[3] Department of Cytometry, Institute for Biotechnology, Technische Universität Berlin, Berlin, Germany

1 Gating for unsorted fixed cells

Figure S1 shows how single cells were gated in the data obtained with the custom-built MAPS flow cytometry setup. This gating is used for Figure 3 in the main manuscript and analysis based thereon.

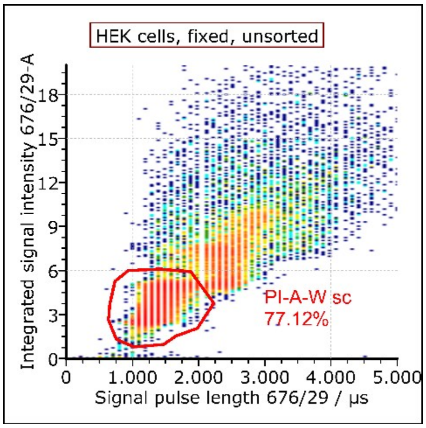

Figure S1: Gating for single cells in fixed HEK cells measured with the MAPS setup. This gating was used for the displays in Figure 3 in the main manuscript.

2 Gating for cluster-sorted fixed cells

Figure S2(a) shows the gating for single cells in fixed HEK cells measured with a FACSCanto II. This gating was used for the graphs and analysis of Figure 6 in the main manuscript. Figure S2(b) shows the fluorescence intensity distribution and cell cycle phase gates of the unsorted sample. It serves as a reference for the data on the cluster-sorted samples in Figure 6 of the main manuscript.

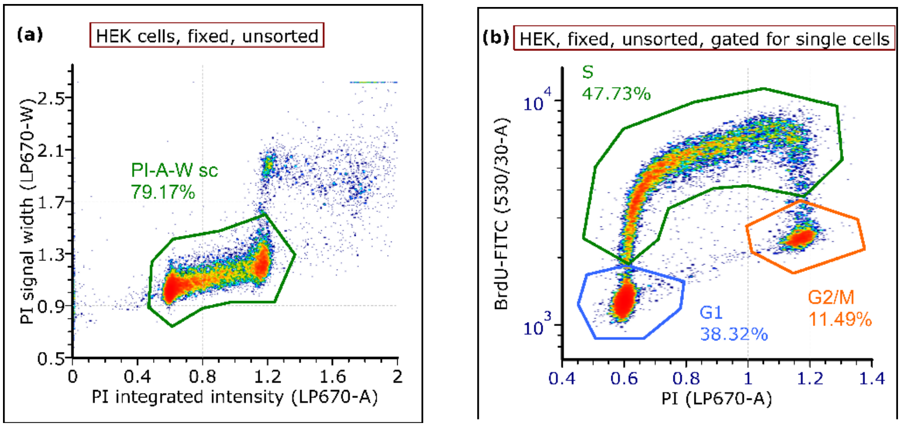

Figure S2: (a) Gating for single cells in fixed HEK cells after cluster-based sorting measured with a FACSCanto II. This gating was used for the displays in Figure 6 in the main manuscript. (b) Fluorescence intensity distribution of unsorted, fixed HEK cells gated for single cells as shown in panel (a). The gating for the three cell cycle phases is shown. This plot serves as a reference for the displays of the cluster-sorted samples in Figure 6 in the main manuscript. The intensity axes are not comparable in absolute terms since re-staining with PI was required after sorting.

3 Gating for scatter-based sort with a conventional cell sorter

The gating hierarchy displayed in Figure S3 was used for the comparative sort of live Jurkat cells with a FACSAria II cell sorter on scattered light parameters (FSC & SSC).

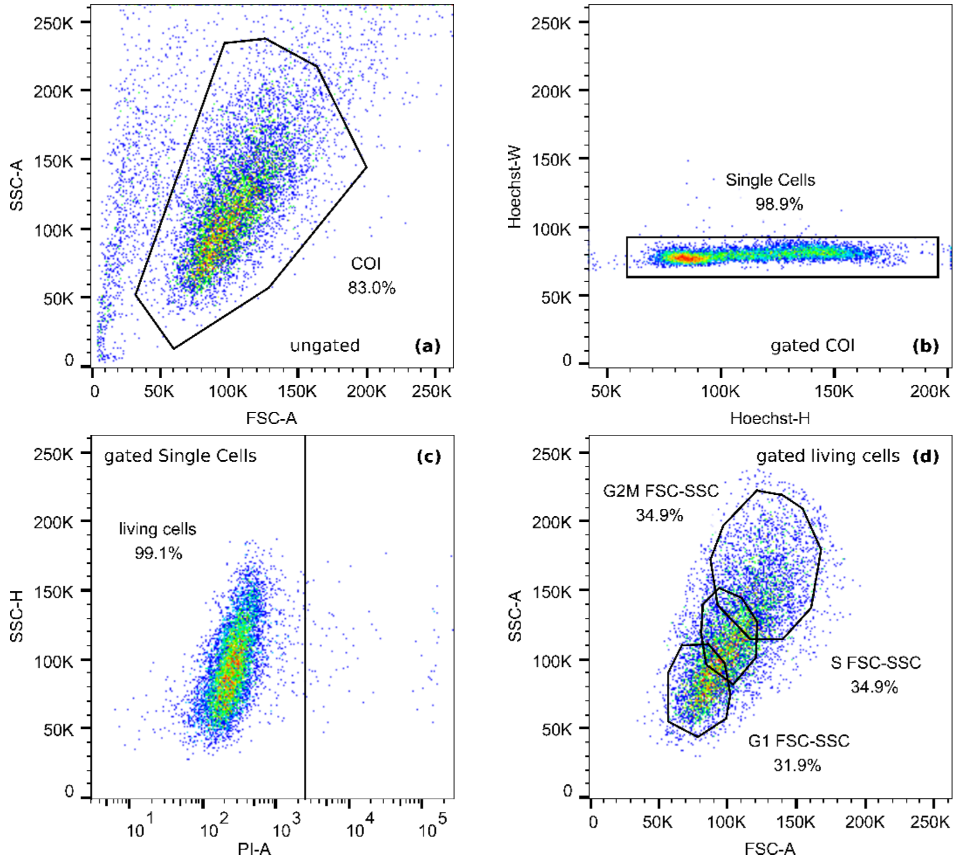

Figure S3: Gating hierarchy for sorting on scattered light parameters with a conventional cell sorter. From (a) to (d), the displayed events are gated as shown in the respective preceding panel. (a) All events with a first clean-up gate. (b) Gating for single cells. (c) Gating on live cells. (d) Gates to sort for cell cycle phases using scattered light parameters.
